# Highly Accurate Digital Processing of Large Stroke Guideway with an Optical Material-Corning Code 7972

**DOI:** 10.3390/ma14143809

**Published:** 2021-07-08

**Authors:** Hanqiang Zhang, Yifan Dai, Tao Lai

**Affiliations:** 1 Laboratory of Science and Technology on Integrated Logistics Support, College of Intelligence Science and Technology, National University of Defense Technology, Changsha 410073, China; k.4526@163.com (H.Z.); dyf@nudt.edu.cn (Y.D.); 2 Hu’nan Key Laboratory of Ultra-Precision Machining Technology, Changsha 410073, China

**Keywords:** optical material guideway, reciprocating processing, magnetorheological, grinding disc polishing, nanometer accuracy

## Abstract

Currently, meter-long guideways rarely achieve an accuracy of dozens of nanometers due to processing difficulties such as the material and the edge effect. In this paper, we focus on this problem and propose a set of optimization processing methods to cope with it. In the grinding stage, a grinding tool is designed to improve the reciprocating processing and address the problem of warping; in the polishing stage, three different processes are compared, and the combination of magnetorheological finishing technology and the polyurethane disc technology process is purposed to reduce the polishing cycle and improve the surface figure accuracy. Moreover, through the combined process of magnetorheological finishing and smoothing, the edge effect and medium- and high-frequency error are essentially suppressed. The meter-long guideway is achieved with an accuracy of dozens of nanometers. Although the sizes of surface A/C and B/D are 1000 mm × 240 mm and 1000 mm × 160 mm, the surface figures are 20.33 nm, 22.78 nm, 39.23 nm and 26.58 nm RMS (Root Mean Square), respectively. The nanometer accuracy guideway is critical to an ultra-precision machine tool. Finally, the *X*-axis straightness of the profile measurement system formed by the guideway reaches 200 nm/600 mm.

## 1. Introduction

The accuracy of machine tool guideways plays an important role in the development of high-precision machining and measuring machines. Moreover, high precision measurement is one of the main driving forces for science and technology, and the interferometer based on the coherent interference effect is one of the most extensively used tools. The conventional guideway cannot be measured by the interferometer due to its unique physical properties that the materials do not possess. In order to use the interferometer during the guideway manufacturing, this article used the optical material-Corning Code 7972 for processing. There are two main factors affecting guideway accuracy: (1) guideway materials, and (2) the processing method.

### 1.1. Guideway Materials

The guideway materials should satisfy the selection criteria, mainly large elastic modulus, low density and good thermal stability [1]. Steel, aluminum alloy, cast iron and other metal materials are common guideway materials.

#### 1.1.1. Steel Guideway

Steel is cheap and is extensively used as guideway material, but the machining accuracy is affected by force and thermal deformation during the processing of steel guideways. Therefore, steel guideways are rarely used on ultra-precision moving platforms [2]. Wang [3] carried out the experimental analysis on trial production of 45 steel inlaid guideways. The friction force of hard steel guideway in Okuma was studied by ANFIS (Adaptive Neuro-Fuzzy Inference System) modeling [4]. The friction of AISI (American Iron and Steel Institute) 1045 guideway between textured surfaces was investigated by reciprocating sliding tests [5,6]. THK (Toughness, High Quality, Know-how) guideway [7] is mainly Austenitic stainless steel and Martensitic stainless steel.

#### 1.1.2. Aluminum Alloy Guideway

High-strength aluminum alloy is a material that has been rapidly developing material in recent years. The advantages of aluminum material are its light weight, high strength, good thermal conductivity and weldability [8]. Carl Zeiss launched the anti-aging aluminum technology to improve guideway stability under varying thermal conditions [9]. Nairong studied the application of boron alloy cast iron to improve the wear resistance of machine tool guideways [10].

#### 1.1.3. Cast Iron Guideway

The effect of boron on the microstructure, mechanical properties and wear resistance was investigated in white cast iron containing different contents [11]. In non-metallic materials, the thermal stability of granite was superior to that of metal materials. Granite guideways are applied in coordinate measuring machines and other precision platforms [12].

#### 1.1.4. New Material Guideway

With the development of material science, some other non-metallic materials, such as alumina ceramics and glass ceramics, have also been gradually applied on guideways [13,14]. For large-scale fine structural alumina matrix ceramic guideway materials, the fracture mechanism and porosity were analyzed by Liu [15]. Liu also studied the effect of load on friction and wear behaviors of the alumina matrix ceramic guideway materials. He found that the wear rate of guideway materials toughened by the diopside and AlTiB master alloys was ten times smaller than the pure alumina [16]. Alumina ceramic guideways were studied earlier by Sodick Corporation and Sumitomo Heavy Industries, and an ultra-precision moving platform was applied [17,18,19,20,21,22,23]. Currently, few optical material guideways have been reported. For traditional slender steel guideways, heat treatment and grinding are applied [24,25], but the guideway accuracy of the process is fundamentally limited by the machining accuracy and measuring accuracy, especially for the large-size guideway.

### 1.2. Processing Method

Traditional optical surface is manufactured by coarse grinding, fine grinding and polishing [26]. The accuracy of coarse grinding is generally in the tens of micrometers. Improvement in surface accuracy is addressed by fine grinding and polishing. Grinding and polishing of long guideways is prone to edge effects; therefore, the sag and warping at the edge of the guideway are not conducive to further improvement in guideway surface accuracy. Based on advanced optical processing technology, a set of combined processes have been proposed to reduce the effect of the edge effect of the elongated guideway and improve the guideway surface accuracy. Jiongkun used the small grinding disc grinding technology of CCOS (Computer Controlled Optical Surfacing) to grind the 480 mm × 75 mm 4Cr13 stainless steel guideway. The guideway surface accuracy was improved from 4 μm RMS (Root Mean Square) to 0.376 μm RMS [27]. With this approach, the edge effect is difficult to remove. Therefore, the magnetorheological finishing technology and CCOS small grinding disc technology were applied for a glass guideways by Guo Meng. The four surfaces of 400 mm × 200 mm × 80 mm K9 glass guideway were below 0.869 μm PV (Peak to Valley) [28,29,30]. The 700 mm × 260 mm aluminum oxide guideway was manufactured by ultra-precision grinding machine tool. The guideway surface accuracy reached 0.78 λ(1 λ = 632.8 nm) PV and 0.1 λ RMS via various abrasive particles and various polishing trajectories [31]. Sumitomo Heavy Industries in Japan used the KSX-815 ultra-precision flat grinder to process a 500 mm × 500 mm workpiece flatness to 1 μm [32].

Non-optical material guideways cannot be measured by interferometer with high accuracy; therefore, the machining accuracy of the guideway cannot achieve a highly accurate surface. Owing to these properties, an accuracy of nanometers is frequently inaccessible by conventional guideways at a large scale. In this paper, we utilize ultra-low expansion glass as the guideway material. This work aims to merge the optical manufacturing method and the properties of ultra-low expansion glass and improves the dynamic characteristics of machine tools and accuracy. The major contributions and innovations of this paper are as follows. A new material is applied on the guideway of the machine tool. The surface of the guideway can be measured by a wavefront interferometer. A new optimization combined processing is proposed to obtain a 22 nm RMS meter-long guideway, using the novel guideway to assemble a 200 nm/600 mm-straightness profilometer.

## 2. Optimization of Combined Processing

Ultra-low expansion glass has been and continues to be a significant material for astronomical applications. With a nominal composition of 7 wt.%TiO_2_ in SiO_2_, Corning Code 7972 (Corning, New York, NY, USA) has a mean room temperature coefficient of thermal expansion of 0 ± 30 ppb/°C with a typical coefficient of thermal expansion range of less than 15 ppb/°C, which are properties vital to the manufacturing of high-resolution optics requiring extreme thermal stability. Therefore, this kind of material can be used as the guideway of the machine tool so that the guideway is not affected by temperature. The initial surface figure of face A is 67.673 λ PV and 8.377 λ RMS.

The theoretical basis of the optical modification process is the Preston equation [33,34]. Because the material removal of the glass guideway has a linear relationship with the dwell time,

(1) ΔH=K·P·V

where Δ*H* is the removal efficiency of the guideway material; *K* is the Preston constant, which is related to factors such as the material of the guideway, the abrasive, the temperature and the performance of the machine tool; *V* is the relative speed of the glass guideway and the rectangular grinding disc; *P* is the positive pressure between the glass guideway and the rectangular grinding disc.

The removal amount of material on the guideway surface *H(x,y)* can be expressed as a convolution between the removal function of a rectangular grinding disc on a glass guide *R*(*x,y*) and dwell time with rectangular grinding disc *T*(*x,y)*,

(2) $$H\left(x,y\right)=R\left(x,y\right)*T\left(x,y\right)=\overset{t}{\underset{0}{\int }}\Delta H\left(x,y,t\right)dt$$

Equation (2) shows that the modification of the guideway can be achieved by controlling the dwell time.

The processing machine tool is a multifunctional 6-axis numerical control machine developed by our own laboratory. The processing range of the machine tool can reach 5 m. The positioning accuracy of the machine is 1.5 μm. The surface figures are measured by two instruments—a coordinate measuring machine and a wavefront interferometer. The Zeiss Accura (Zeiss, Heidenheim, Germany) coordinate measuring machine has an accuracy of [2.2 + L/300] μm, and the L is the measuring position. The Zygo VeriFire 24″ interferometer (Zygo, Middlefield, CT, USA) has an accuracy of 0.63 nm RMS. The coordinate measuring machine is used for rough processing, and the interferometer is used for finish processing.

### 2.1. Rectangular Grinding Disc Process

#### 2.1.1. Removal of the Edge Effect

The profile error of the guideway has a very important impact on the straightness of the aerostatic guideway. The guideway surface accuracy is limited by the “edge effect” that occurs during processing. This section introduces a reciprocating linear processing method [35]. The initial surface figure has a 67.673 λ PV (1 λ = 632.8 nm) and an 8.377 λ RMS.

**Figure 1:**
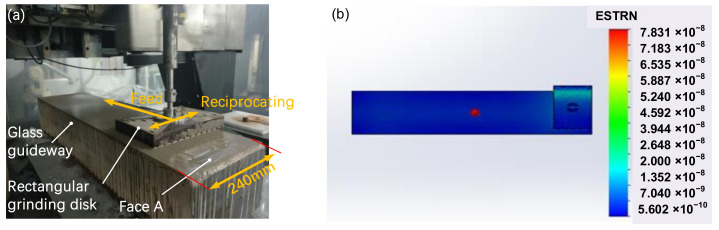
(**a**) Reciprocating of grinding disc reciprocating. (**b**) Simulation of stress distribution.

The rectangular grinding disc reciprocating can be referred to as computer controlled optical surfacing reciprocating motion processing. The principle is that the rectangular grinding disc performs sinusoidal trajectory processing, which can decompose the movement into two vertical movements; the rectangular grinding disc and the guideway have the same width. The motion decomposition diagram is illustrated in Figure 1a. The edge pressure is larger than the middle area, and its stress simulation distribution is shown in Figure 1b. The simulation parameters are as follows. The simulation software is SolidWorks SimulationXpress (Dassault Systemes, Waltham, MA, USA). The grid is selected standard mode, the overall size is 47.3 mm and the tolerance is 2.35 mm. The ambient temperature is set at 22 °C. Leakage distance in the width direction is 10 mm, there is no leakage distance in the length direction, the weight of the disc is 6 kg, and the machine pressure on the disc is 0.1 MPa. Therefore, the warpage can be modified by the disc. According to the dwell time difference of the lengthwise processing, the high points are removed to improve the surface figure.

**Figure 2:**
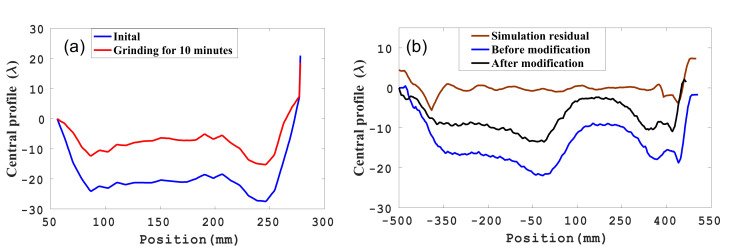
(**a**) Central profile changes along the width direction. (**b**) Central profile changes along the length direction.

Before processing, the face A of the guideway has significant warpage along the width direction, as shown by the blue line in Figure 2, and the straightness value along the middle width direction (central profile) of the guideway is 48.7 λ. To verify that the rectangular grinding disc reciprocating linear processing can remove the edge effect, the grinding process experiment was performed on the face A of the guideway. The experimental parameters were as follows: the cast iron grinding disc weighed 6 kg, using W20 diamond powder as the abrasive; the machine swing speed was 120 r/min (the swing period is 0.5 s); the swing amplitude was 30 mm; and the machine feed speed was 200 mm/min. After processing with the feed speed for 10 min, the central profile of the processed guideway is shown in Figure 2a. The result shows that the rectangular grinding disc reciprocating linear processing can remove the edge effect.

#### 2.1.2. Modification along the Length Direction

It can be seen that the rectangular grinding disc reciprocating linear processing can remove the edge effect of the glass guideway from Figure 2a, but the application of the specific modification ability on the meter-long glass guideway needs to be verified.

As shown in Figure 2b (the blue line), the central profile of the glass guide before the modification was 22.37 λ. Due to the movement characteristics of the rectangular disc, the removal function *R*(*x*,*y*) and the guideway were simplified into a one-dimensional model *R*(*x*) *H*(*x*). The efficiency was 1.5 μm/min. The profile convergence graph obtained by simulation is shown in Figure 2b (the red line).

**Figure 3:**
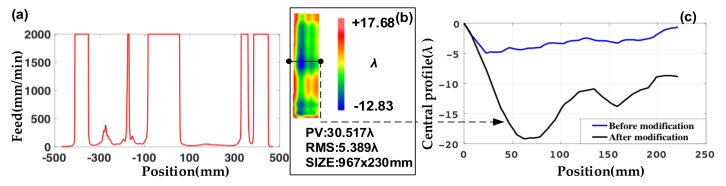
(**a**) Simulation feed speed at different positions. (**b**) Surface figure after modifications. (**c**) Central profile changes before and after modification.

For the case in which the maximum feed of the machine tool is set at 2000 mm/min, the distribution of simulation feed speed is shown in Figure 3a. The processed surface figure is shown in Figure 3b (the result is measured by a coordinate measuring machine with an accuracy of [2.2 + L/300] μm, and the L is measuring range), and the lateral central profile of the guideway through modification is shown in Figure 3c (the black line).

However, there is a serious edge effect in the width direction. According to the processed result and the speed profile of the simulation, it is known that when the feed is 2000 mm/min, the reciprocating time period is too short to remove the edge effect. The central profile increased from 4.96 λ to 19.19 λ, as shown in Figure 3c. Details on solving the increase by optimizing the grinding disc are presented in the next section.

#### 2.1.3. Optimization of the Grinding Disc

The design of the rectangular grinding disc considers two aspects: one of them is processing efficiency and the other is processing accuracy. The rectangular disc with a width of 240 mm is convenient to process and locate, and the center of the resident function is aligned with the center of the guideway surface.

**Figure 4:**
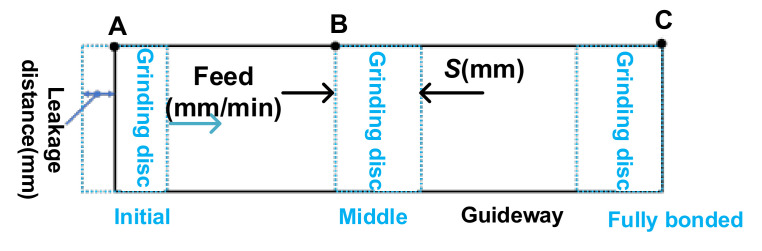
Dwell point of the guideway.

According to the Preston equation, the deterministic removal is based on the linear rule that the removal amount is proportional to the pressure and feed. In order to reduce the path difference of the grinding tool when processing at three points, A, B and C (shown in Figure 4), Point A and point C are the edge points of the guideway and point B is the point of the middle part of the guideway; the pressure is assumed to take the same value for each point between the rectangular grinding disc and the guideway; the leakage distance is equal to the width of disc; the dwell time of the rectangular grinding disc is the same at points A, B and C; and the removal amount of the three points is the same. In reality, there is a leakage at point A/C, and the dwell time of A/C is shorter than that of B. The particle width of the grinding disc is further reduced to decrease the dwell time difference between the A/C and B.

During the processing of the guideway, the processing model is simplified into a one-dimensional model. The width of the rectangular grinding disc is the diameter of the removal function. The modification of the guideway is realized by the different dwell times of the rectangular grinding disc. In the actual modification, the larger the removal function size, the worse the surface modification ability (accuracy) [36] but the faster the processing. To account for the tradeoff between time and accuracy, half of the longitudinal spatial wavelength of the central profile of the guideway (224 mm) is taken as the length of the rectangular disc. Therefore, the size of the disc is set at 112 mm × 240 mm.

In order to compare the stress changes of the grinding disc before and after optimization, COMSOL (Comsol, Stockholm, Sweden) is used to simulate the stress distribution of the grinding disc on the guideway. There are three cases:

(1) The initial stress distribution of the grinding disc on the guideway;
(2) The stress distribution when the new grinding disc is fully bonded to the guideway;
(3) The stress distribution when the grinding disc is located in the middle of the guideway.

Generally, the leakage distance is about 1/3 of the size of the grinding tool. The size of the new grinding disc is designed to be 112 mm × 240 mm, so the leakage is 40 mm; the size of the old grinding disc is 160 mm × 240 mm, and the leakage is 50 mm.

**Figure 5:**
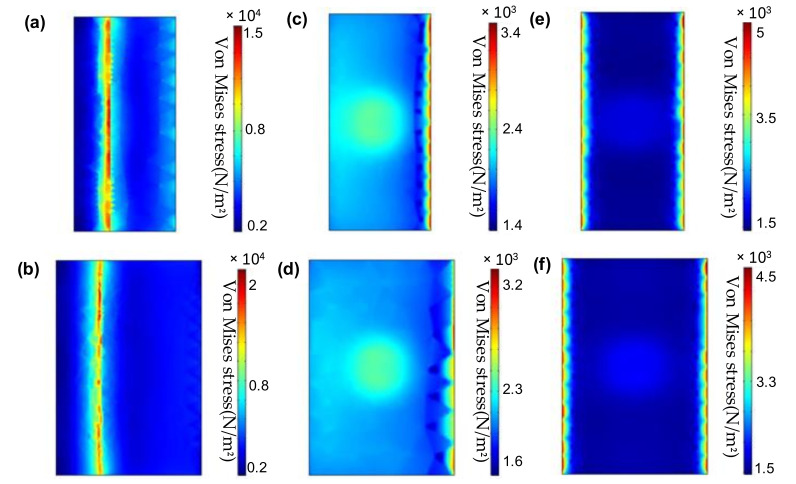
(**a**) Initial stress distribution of new grinding discs. (**b**) Initial stress distribution of old grinding discs. (**c**) Stress distribution when the new grinding disc is fully bonded to the guideway. (**d**) Stress distribution when the old grinding disc is fully bonded to the guideway. (**e**) Stress distribution when the new grinding disc is in the middle of the guideway. (**f**) Stress distribution when the old grinding disc is in the middle of the guideway.

**Figure 6:**
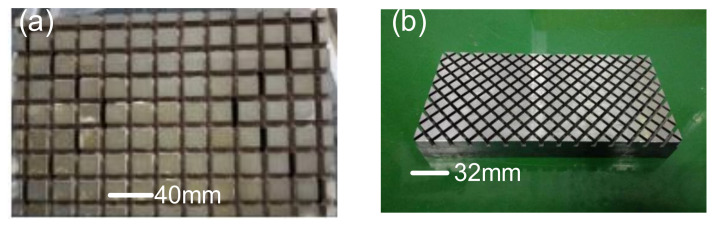
(**a**) Front of old grinding disc. (**b**) Front of new grinding disc.

By comparing the three distributions of the new and old grinding disc guides, as shown in Figure 5. (Simulation parameters: The simulation software is COMSOL). The surface boundary constraint is selected as the boundary condition. The mesh shape is triangular mesh and the mesh size is 20 mm. The ambient temperature is set at 22 °C (a) and (b) shows the simulation of position A in Figure 4. The leakage distances are 40 mm and 50 mm respectively; (c) and (d) are the simulations of position C in Figure 4; (e) and (f) are the simulation of position B in Figure 4. The machine pressure on the disc is 0.1 MPa.) It was found that the old grinding disc has a higher stress in the initial position, while in the remaining two cases, the new grinding disc has higher stress. This phenomenon is caused by the larger leakage of the old grinding disc in the initial position, while the new grinding disc is heavier than the old grinding disc, so the removal efficiency is higher than that of the old grinding. The physical disc is shown in Figure 6.

#### 2.1.4. Optimization of the Speed

When feed speed is kept constant, the reciprocating processing method can remove the edge effect and improve the surface. The guideway surface figure can be simplified to a one-dimensional error during the modification. The dwell time and the corresponding feed distribution are solved by the removal efficiency of the grinding disc and longitudinal central profile of the guideway.

For the old grinding disc during the processing, when the swing speed *n* is 120 r/min, the width of old grind disc *S* is 160 mm and the maximum feed speed *F*_1_ is 2000 mm/min, the dwell time *T*_1_ at point B is:

(3) $$T_{1}=\frac{S}{F_{1}}$$

(4) $$c_{1}=nT_{1}=n\frac{S}{F_{1}}$$

Therefore, the grinding disc reciprocates for 9.6 cycles, the trajectory density is sparse and the material is not uniformly removed, resulting in the occurrence of warping.

When the minimum feed speed *F*_2_ is 100 mm/min, the dwell time *T*_2_ of point B is 1.6 min, the grinding disc swings 192 cycles, the trajectory is dense, the material is removed uniformly, and the surface accuracy is good:

(5) $$T_{2}=\frac{S}{F_{2}}$$

(6) $$c_{2}=nT_{2}=n\frac{S}{F_{2}}$$

The edge effect is removed obviously when the feed speed is 400 mm/min. In order to ensure that the abrasive does not lose too much during grinding, the highest feed rate is set at 800 mm/min and the swing speed is set at 240 r/min, so that the swinging trajectory of the grinding disc is the same as the feed speed of 400 mm/min and swing speed is 120 r/min.

(7) $$c\geq nT=120r/min\times \frac{160mm}{400mm/min}=48r$$

Equation (7) is an empirical threshold formula. The edge effect can be removed when the lateral grinding disc is 160 mm and the cycles of swing are 48.

**Figure 7:**
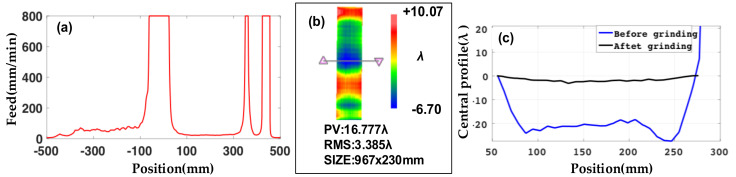
(**a**) Simulation feed speed at different positions. (**b**) Surface figure after modification. (**c**) Central profile changes before and after modification.

The feed distribution of the new grinding disc at each point on the guideway is simulated and shown in Figure 7a. With the new disc and optimization parameters, the PV of the surface figure is 16.77 λ after grinding, as shown in Figure 7b. The width central profile is 3.19 λ after grinding as shown in Figure 7c. All the results prove the feasibility of the optimization scheme. (Simulation parameters before optimization: old disc, swing speed is 120 r/min, feed speed is 2000 mm/min, dwell time is 0.08 min, grinding disc reciprocates is 9.6 r. Simulation parameters after optimization: disc, swing speed is 120 r/min, feed speed is 100 mm/min, dwell time is 1.6 min, grinding disc reciprocates is 192 r.)

### 2.2. Combined Process

The coordinate measuring machine does not usually meet the high surface accuracy such as one with a PV better than 1 μm. The 24-inch Zygo (Zygo, Middlefield, CT, USA) wavefront interferometer measurement can achieve nanometer accuracy. The interference measurement method requires the measured workpiece to have a smooth surface and a glossy mirror surface. The surface of the glass guideway is polished, and the processing is guided by advanced optical measurement methods. This section introduces three different polishing combined processes. an optimal combined process is chosen for the processing of meter-long glass guideways.

#### 2.2.1. MRF and CCOS Small Disc Combined Polishing

Corning 7972 glass (Corning, New York, NY, USA) is a hard and brittle material with low fracture toughness. The surface is fragmentation-manufactured by the ordinary cast iron grinding disc. The surface and subsurface of the guideway after processing crack and produce defects. Magnetorheological finishing uses the characteristics of rheological properties of magnetic materials such as iron powder in the polishing fluid [37] to polish the surface of meter-long glass guideways. In the processing area, due to the effect of high-intensity gradient magnetic field, the viscosity and hardness of MR fluid rise and are transformed into Binham medium, and a “flexible polishing film” is formed [38,39,40], so the plastic removal of shear stress is achieved on the guideway surface. Due to the removal characteristics of the MRF (Magnetorheological Finishing) technology, the surface roughness of the glass guideway can be improved, and a high-quality surface can be obtained [41]. However, the MRF technology has a small removal function, and the mid- and high-frequency errors will occur on the surface. The existing methods to eliminate mid- and high-frequency errors are the CCOS processes. Therefore, in this section, the errors are eliminated by MRF and CCOS small grinding disc smoothing technology, so that the meter-long guideway surface can be connected from three-coordinate measurement to optical interference measurement without damaging the surface figure.

**Figure 8:**
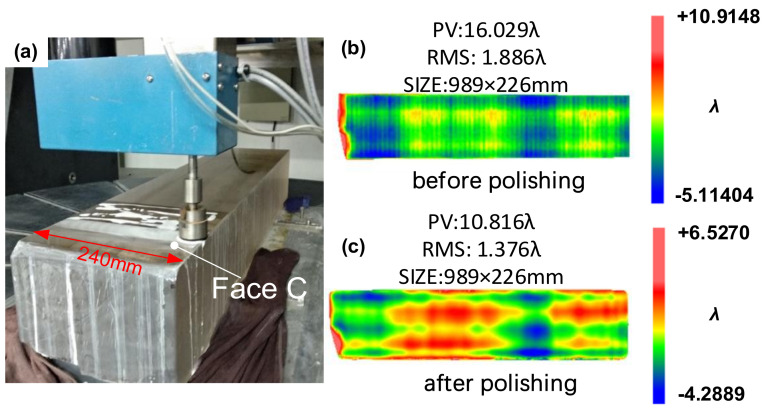
(**a**) Small grinding disc polishing. (**b**) Surface figure before grinding. (**c**) Surface figure after grinding.

The guideway is polished by the small disc as shown in the Figure 8a. The initial figure of the face C of the guideway is shown in Figure 8b (measured by the coordinate measuring machine), and the PV of the surface figure before polishing is 16.029 λ. The parameter setting of magnetorheological finishing and small disc polishing is listed in Table 1. Figure 8c shows the C surface figure after 27 h of uniform removal by magnetic rheology and 8 h of CCOS small grinding disc polishing (measured by the coordinate measuring machine).

Comparing the surface figure initial and polishing, the process of magnetorheological finishing and small grinding disc smoothing can achieve the polishing of the guideway, and the surface figure has a convergence from 16.029 λ to 10.816 λ. According to the entire surface figure, it can be seen that the surface figure along the width direction is discrete. This is because the size of the removal function of the combined polishing (the magnetorheological finishing and the small grinding disc) is relatively small.

#### 2.2.2. CCOS Large Disc Combined Polishing

The grinding disc has a large removal function, which can increase the removal efficiency. The experiment was performed with a *ϕ*200 mm polyurethane polishing disc. The experimental surface figure is face A. The data before polishing are shown in the Figure 7b, and the processing parameters are listed in Table 2.

**Figure 9:**
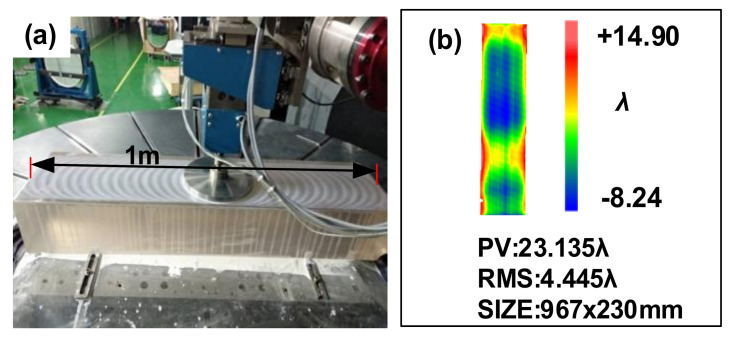
(**a**) *ϕ*200 mm polyurethane disc polishing (**b**) Surface figure after polishing.

As shown in Figure 9a, the guideway is processed by the *ϕ*200 mm polyurethane disc. A comparison of Figure 9b (measured by the coordinate measuring machine) and Figure 7b reveals the deteriorated surface accuracy after processing for 87 min, with the PV value changed from 16.777 λ to 23.135 λ. The lateral profile was accompanied by obvious warping, and the guideway became slightly bright after processing. There are two reasons for this result:

(1) The movement of the CCOS circular polishing disc to the edge of the guideway causes a great change in edge pressure, which will cause the deviation of the edge material removal efficiency from the theoretical value, and the edge effect of CCOS processing is generated [42].
(2) According to the Preston equation, the removal amount is positively related to the pressure. During the processing, the pressure distribution is uneven on the round polishing disc, and there is no leakage; therefore, the warping is generated.

**Figure 10:**
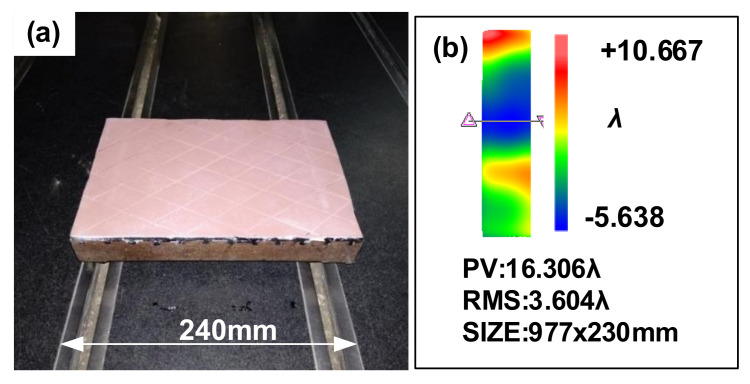
(**a**) Polyurethane rectangular disc polishing. (**b**) Surface figure after polishing.

In Section 2.1, the linear reciprocating processing of rectangular discs can remove the edge effect; therefore, it was proposed to combine the CCOS round grinding disc technology with the rectangular disc technology. A 240 mm × 160 mm polyurethane rectangle disc (mass: 8 kg) was applied for processing as shown in Figure 10a, with the specific experimental parameters listed in Table 3.

The optimization process was obtained to improve the surface figure accuracy by processing: *ϕ*200 mm polyurethane disc was polished for 87 min; then, the 240 mm × 160 mm polyurethane rectangular disc was processed for 27 min; finally, the *ϕ*200 mm polyurethane disc was polished for 435 min again. The processing result is shown in Figure 10b (the result is measured by a Zygo VeriFire 24″ interferometer with an accuracy of 0.63 nm RMS (*k* = 2)), and the edge effect is removed.

The experimental results show that the CCOS large disc combination polishing technology has a fast-polishing effect. The warped edge along the width direction can be controlled by the rectangular disc. The central profile along the width direction after processing is 0.64 λ. However, the profile accuracy along the length direction cannot reach a higher accuracy with the combined polishing, and the polished surface figure has no obvious convergence.

#### 2.2.3. MRF and CCOS Large Disc Polishing

Magnetorheological finishing is a kind of flexible polishing. Due to the unique shear removal characteristics, it can repair surface and sub-surface damage, obtain excellent surface quality, and achieve conformal polishing. If the MRF is used alone, the processing speed of the meter-long guideway cannot be improved. The rapid polishing is applied with a large CCOS disc, but the accuracy of the polished surface figure is not high. Therefore, a process is proposed: the MRF is used as a pre-polishing to improve the surface quality of the guideway, and the CCOS is applied for rapid polishing.

**Figure 11:**
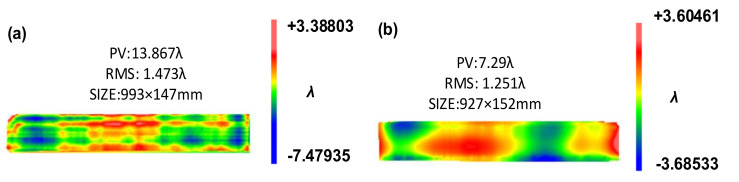
(**a**) Surface figure before polishing. (**b**) Surface figure after polishing.

In order to verify the process, the face D of the guideway was selected as the experimental object. The initial surface figure is shown in Figure 11a (measured by the interferometer): the PV is 13.867 λ, and the parameters of the magnetorheological processing are listed in Table 4.

After about 6 h of uniform removal of the MRF, the guideway was polished by *ϕ*150 mm polyurethane disc; the parameters are listed in Table 5.

After 12 h of polishing, the guideway surface was completely polished. Figure 11b (measured by the interferometer) shows the polished surface figure. The central profile in the width direction was 1.28 λ, and the central profile along the length direction was 5.08 λ. The surface PV after polishing dropped from 13.867 λ to 7.029 λ, and the surface figure was greatly improved, which proves that the combination of magnetorheological finishing and CCOS large disc polishing process achieves conformal polishing and rapid polishing.

## 3. Final Surface Figures and Application

**Figure 12:**
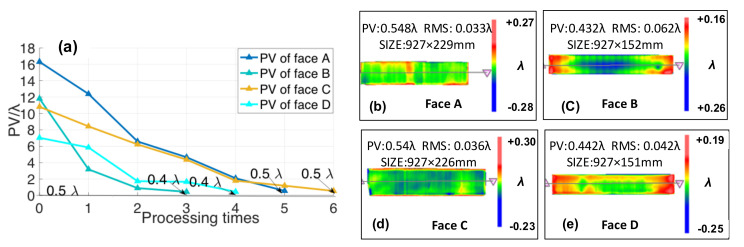
(**a**) PV changes of four surface figures. (**b**) Final surface figures of Face A. (**c**) Final surface figures of Face B. (**d**) Final surface figures of Face C. (**e**) Final surface figures of Face D.

Therefore, the optimized combination process is obtained: the rectangular grinding disc reciprocating processing method is applied for grinding; the magnetorheological is treated as the pre-polishing, and the guideway is polished by *ϕ*150 mm polyurethane disc; and finally, the guideway is smoothened by the MRF and *ϕ*100 mm asphalt disc. The PV changes of four surface figures are shown in Figure 12a during the processing, and the final surface figures are shown in Figure 12b–e (measured by the interferometer). The PV of four surface figures is smaller than 0.5 λ, and the RMS is 23 nm.

**Figure 13:**
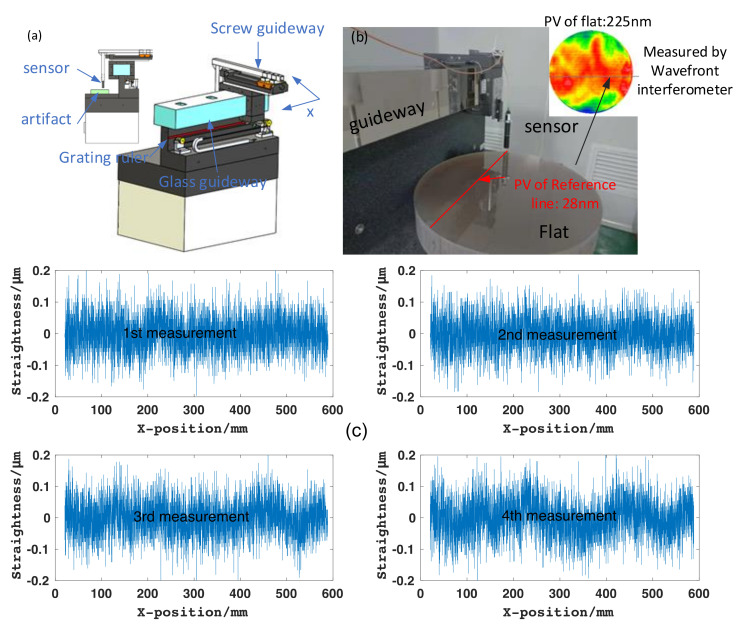
(**a**) Glass-guideway profilometer. (**b**) Measurement setup. (**c**) Four straightness meas-urements.

The profilometer with the glass guideway is shown in Figure 13a. The straightness of the measurement system was calibrated with the optical flat method. The straightness was measured with the ISO 230-1: 2012 method (flat method) [43]. The flat is an optical standard part, and its PV value is 225 nm and its RMS is 12.03 nm; the reference line on the flat is 28 nm. The sensor is a nano-displacement sensor (Stil-CL2, STIL, Aix-en-Provence, France)) with a resolution of 8 nm. The measurement setup is depicted in Figure 13b. In the experiment, the sampling period was set as 0.025 s, and the travel speed was 300 mm/min. The measurement results are shown in Figure 13c. The movement *X*-axis was formed by the glass guideway after four repeated measurements; the results are 213 nm/595 mm, 214 nm/595 mm, 205 nm/595 mm and 217 nm/595 mm.

## 4. Conclusions

The accuracy of a precision machine is limited by the precision guideway, and new materials, advanced processing and measurement methods are the way to realize the high precision. This study was conducted using the optical material (Corning 7972) and one set of advanced processing to manufacture the guideway. Through optical processing and interferometry, a glass guideway was obtained with a higher accuracy than general mechanical processing. Therefore, the accuracy of an ultra-precision machine was improved by its higher accuracy guideway. The following conclusions have been drawn from the study of guideway processing technology:

(1) MRF and CCOS small-disc combined polishing, CCOS large-disc combined polishing, and MRF and CCOS large-disc polishing were compared to obtain the optimization process.
(2) The optimization process was as follows: First, the guideway was reciprocating-processed with a rectangular grinding disc. Second, the magnetorheology was treated as the pre-polishing. Third, the guideway was polished by *ϕ*150 mm polyurethane disc. Finally, the guideway was smoothened by an MRF and *ϕ*100 mm asphalt disc.
(3) According to the combined process, a 23 nm manufacturing accuracy of the meter-long guideway was achieved.
(4) The straightness of the profilometer was reached at 200 nm/600 mm based on the glass guideway, which was higher in accuracy than traditional guideways.

However, the one-dimensional theoretical model of reciprocating processing needs to be further improved in the two-dimensional model. It is also necessary to carry out speed compensation through the pressure distribution of the guideway and the processing stroke of the edge point in order to carry out quantitative research.

## Figures and Tables

**Table 1:** Parameter of magnetorheological finishing and small grinding disc polishing.

MRF Wheel		Small Grinding Disc	
Viscosity	213	Diameter of wheel	340 mm
Flow rate	180 L/h	Rotation speed	75 r/min
Abrasive	Cerium oxide W3	Diameter of the dis	40 mm
Feed speed	1200 mm/min	Rotation speed	75 r/min
Magnetic field intensity	8 A	Distance between disc center and guideway edge	8 mm

**Table 2:** Parameter of CCOS large polishing disc.

Air Pressure	Rotating Speed	Distance between Plate Center and Guideway Edge	Feed Rate	Abrasive
0.1 MPa	75 r/min	25 mm	2000 mm	cerium oxide W3

**Table 3:** Parameter for reciprocating linear polishing of rectangular disc.

Machine Speed	Swing	Feed	Abrasive
120 r/min	30 mm	10 mm/min	cerium oxide W3

**Table 4:** Magnetorheological processing parameters.

Flow	Viscosity	Diameter of Polishing Wheel	Rotating Speed	Magnetic Field Strength	Feed
180 L/h	213	*ϕ*340	75 r/min	8 A	1200 mm/min

**Table 5:** Polyurethane disc processing parameters.

Air Pressure	Rotating Speed	Distance between Plate Center and Guideway Edge	Feed	Abrasive
0.1 MPa	75 r/min	15 mm	800 mm/min	cerium oxide W3

## Data Availability

The data presented in this study are available on request from the corresponding author.
